# Supplementary material for: Efficient and stable sky-blue delayed fluorescence organic light-emitting diodes with CIEy below 0.4
Source: Nat Commun. 2018 Nov 28;9:5036. doi: 10.1038/s41467-018-07482-6 (PMC6261989; doi:10.1038/s41467-018-07482-6)
Supplement: Supplementary file 1 — Supplementary Information [file 41467_2018_7482_MOESM1_ESM.pdf]

Supplementary Information

Efficient and Stable Sky-Blue Delayed Fluorescence Organic Light-Emitting Diodes with CIE_y below 0.4

*Chin-Yiu Chan, Masaki Tanaka, Hajime Nakanotani, Chihaya Adachi**

Dr. C.-Y. Chan, Mr. M. Tanaka, Dr. H. Nakanotani, Prof. C. Adachi

Center for Organic Photonics and Electronics Research (OPERA), Kyushu University, 744 Motooka, Nishi, Fukuoka 819-0395, Japan

E-mail: adachi@cstf.kyushu-u.ac.jp

Prof. C. Adachi

International Institute for Carbon Neutral Energy Research (WPI-I2CNER), Kyushu University, 744 Motooka, Nishi, Fukuoka 819-0395, Japan

E-mail: adachi@cstf.kyushu-u.ac.jp

Content

Page

Characterization and NMR spectra……………………………………………………3
Detailed photophysical, electrochemical and thermal measurements………………11
OLED performances and device stability…………………………………………. 19
Summary of stable phosphorescence and TADF OLEDs……………………………31
References……………………………………………………………………………..34

Supplementary Figures

NMR

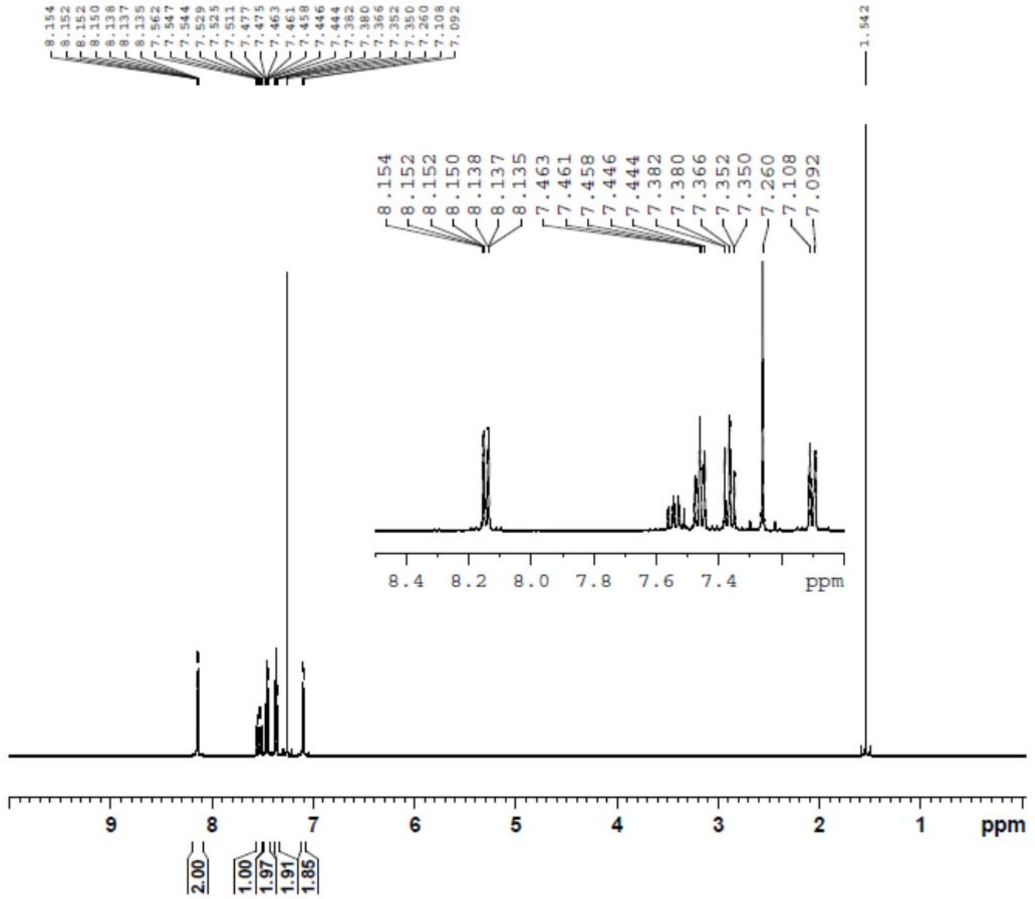

Supplementary Figure 1. ^{1}H NMR spectrum of 2-(9H-Carbazol-9-yl)-3,5,6-trifluorobenzonitrile.

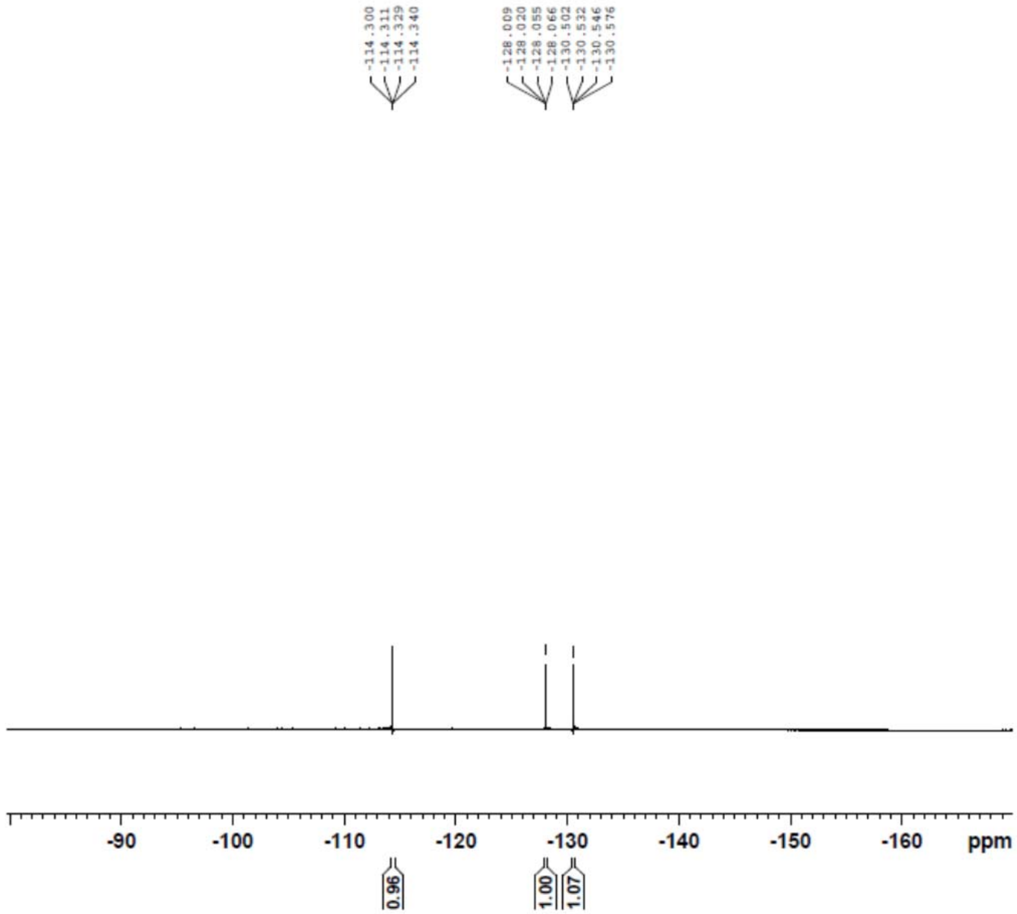

Supplementary Figure 2. ^{19}F NMR spectrum of 2-(9H-Carbazol-9-yl)-3,5,6-trifluorobenzonitrile.

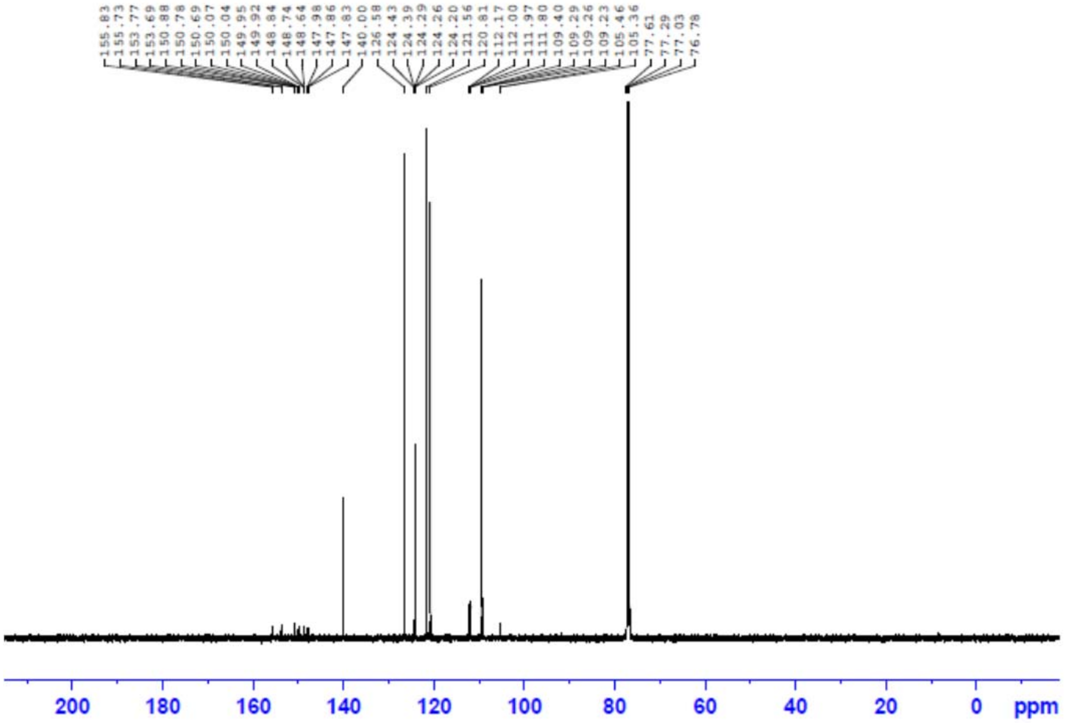

Supplementary Figure 3. ^{13}C NMR spectrum of 2-(9H-Carbazol-9-yl)-3,5,6-trifluorobenzonitrile.

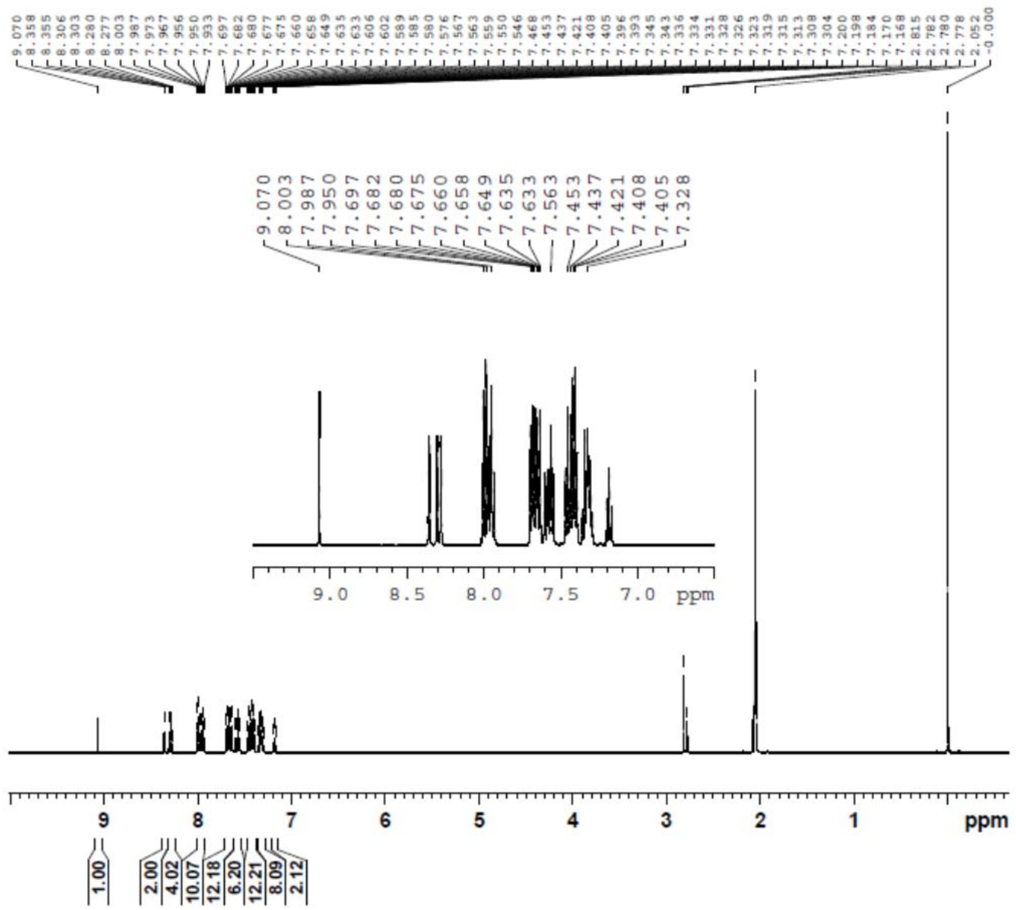

Supplementary Figure 4. ^{1}H NMR spectrum of **3Ph$_2$CzCzBN**.

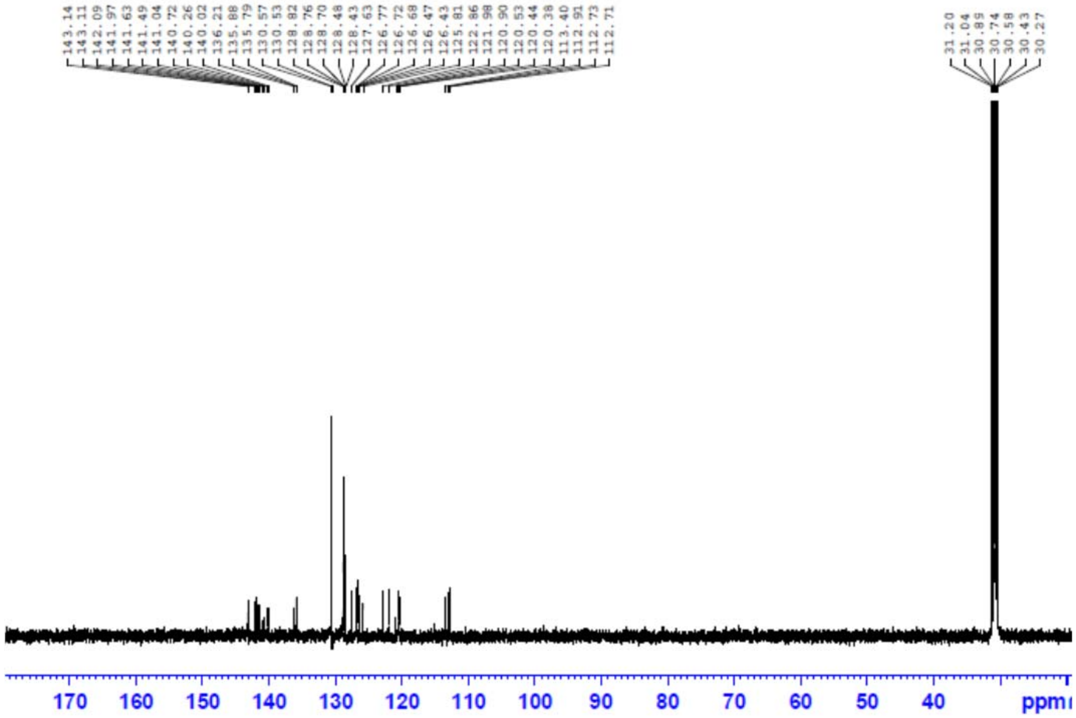

Supplementary Figure 5. ^{13}C NMR spectrum of **3Ph$_2$CzCzBN**.

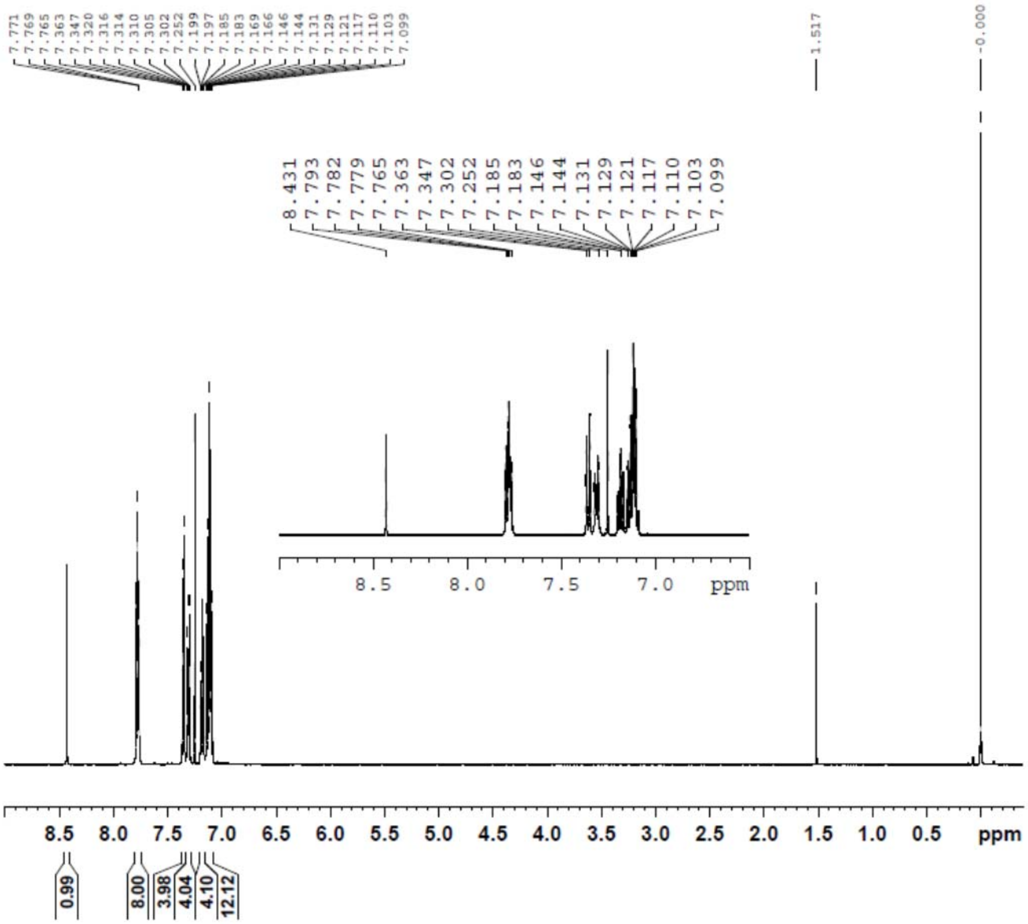

Supplementary Figure 6. ^{1}H NMR spectrum of **4CzBN**.

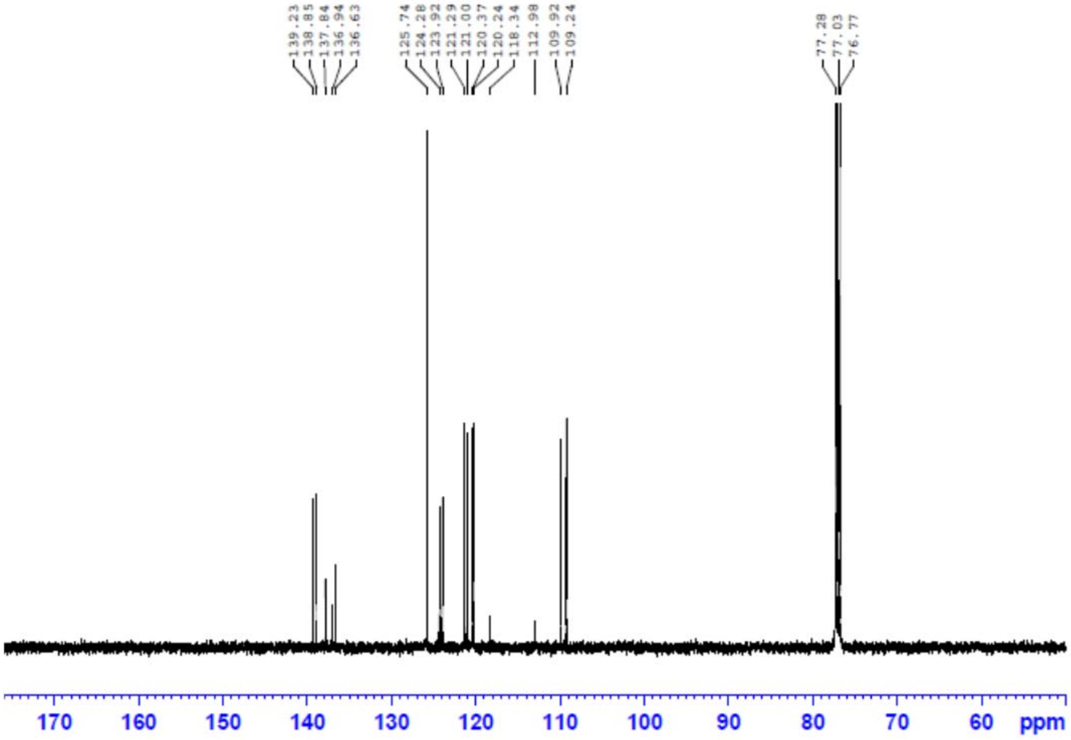

Supplementary Figure 7. ^{13}C NMR spectrum of **4CzBN**.

Purity

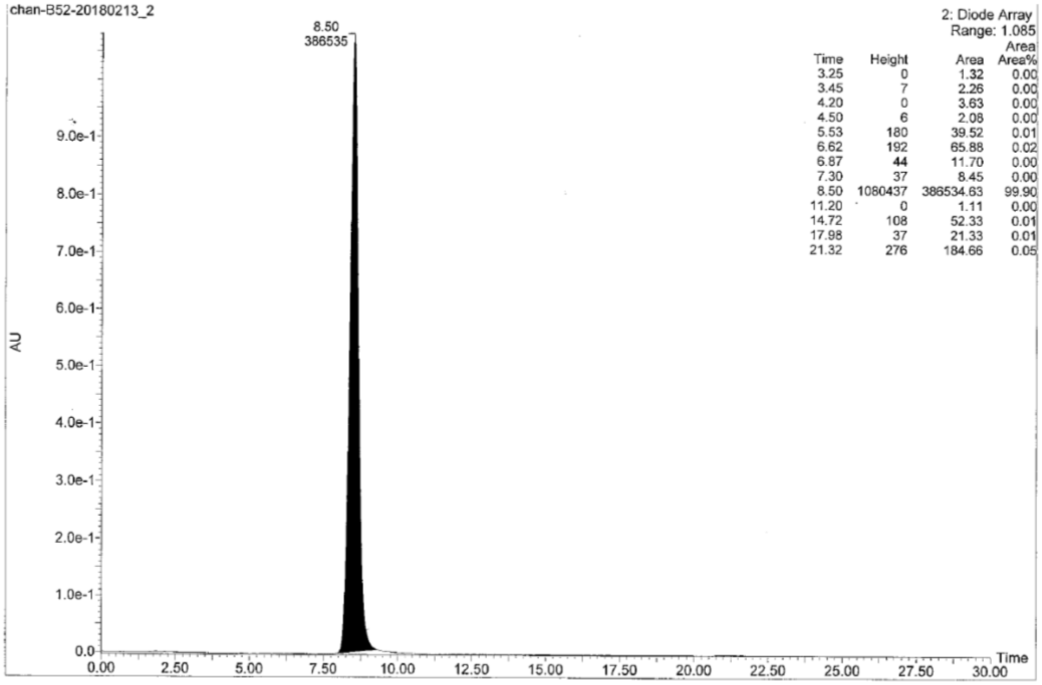

Supplementary Figure 8. Purity of **3Ph$_2$CzCzBN** (99.90%) by HPLC.

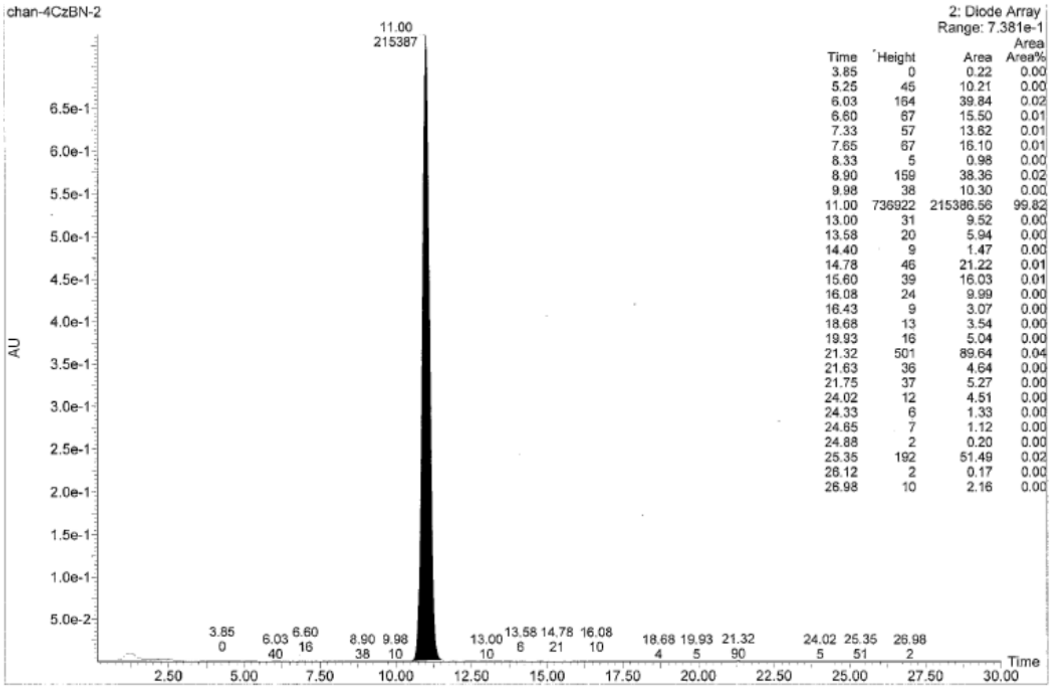

Supplementary Figure 9. Purity of **4CzBN** (99.82%) by HPLC.

Detailed photophysics of 3Ph$_2$CzCzBN and 4CzBN.

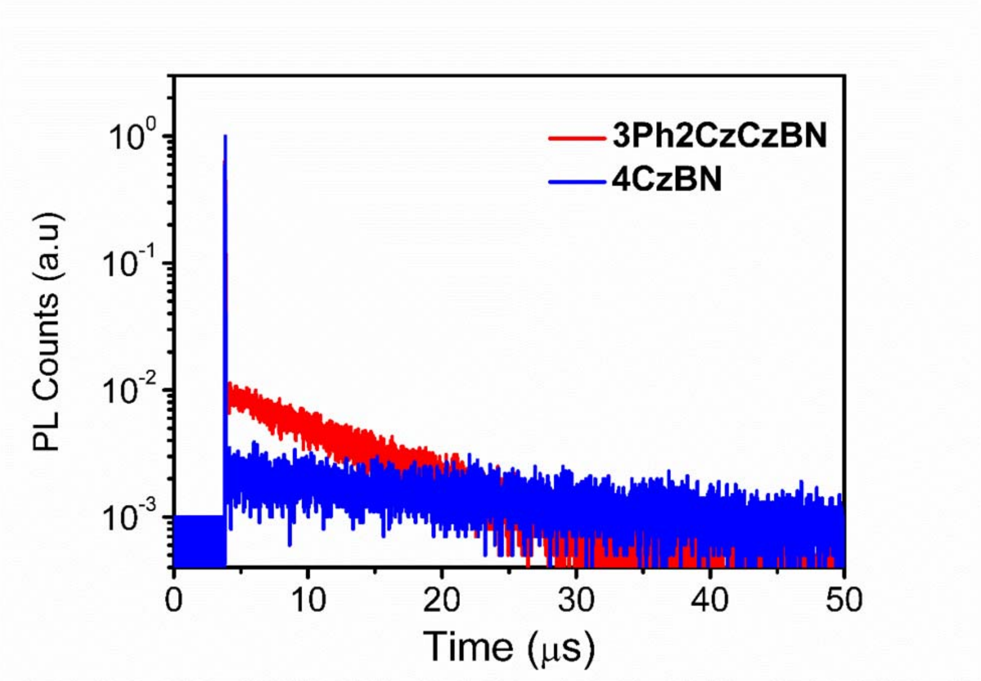

Supplementary Figure 10. Transient decay profiles of **3Ph$_2$CzCzBN** and **4CzBN** in degassed toluene (10^{-5} M) at room temperature.

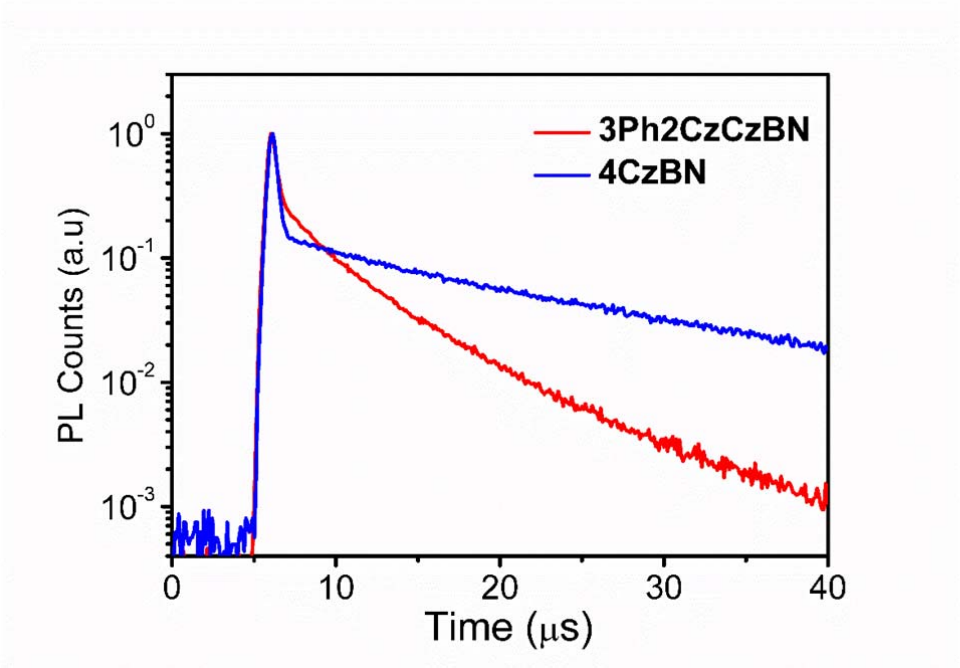

Supplementary Figure 11. Transient decay profiles of 15 wt % of **3Ph$_2$CzCzBN** and **4CzBN** doped in mCBP at room temperature under argon.

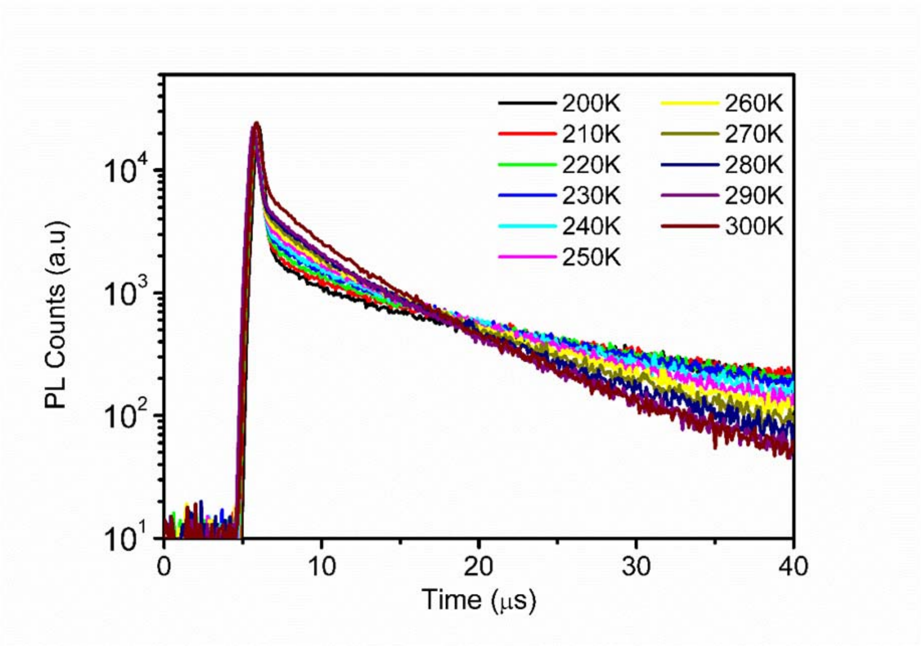

Supplementary Figure 12. Transient decay profiles of 15 wt% **$3Ph_2CzCzBN$** doped in mCBP from 200 to 300K.

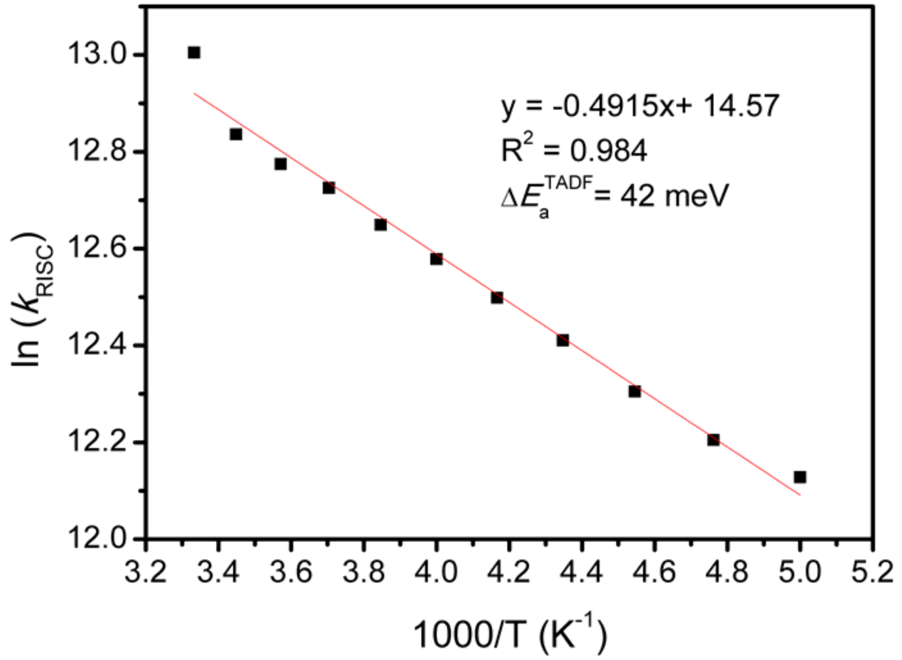

Supplementary Figure 13. Arrhenius plot of 15 wt % **$3Ph_2CzCzBN$** doped in mCBP. The slope equals to -ΔE_a^{TADF}/ 1000 k_B, where k_B ~ 8.62 x 10^{-5} eV K^{-1}.

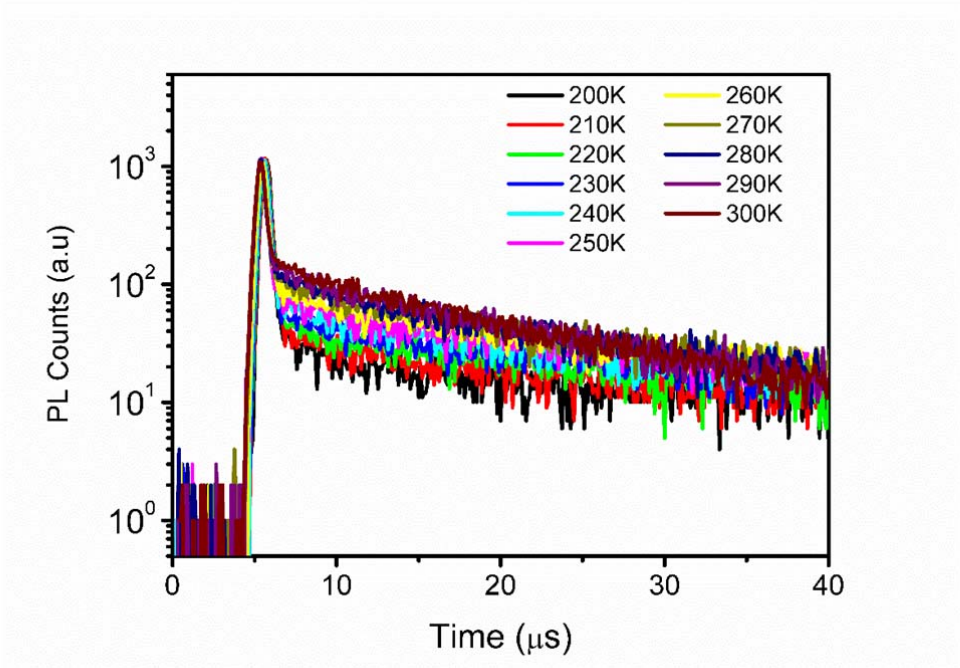

Supplementary Figure 14. Transient decay profiles of 15 wt % **4CzBN** doped in mCBP from 200 to 300K

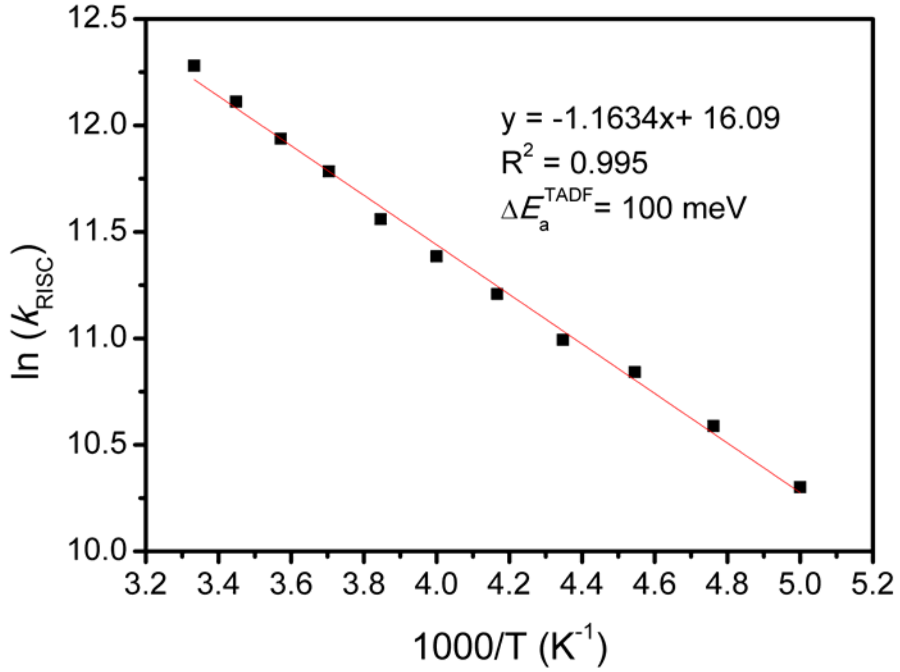

Supplementary Figure 15. Arrhenius plot of 15 wt % **4CzBN** doped in mCBP. The slope equals to -ΔE_a^{TADF}/ 1000 k_B, where k_B ~ 8.62 x 10^{-5} eV K^{-1}.

Cyclic voltammetry

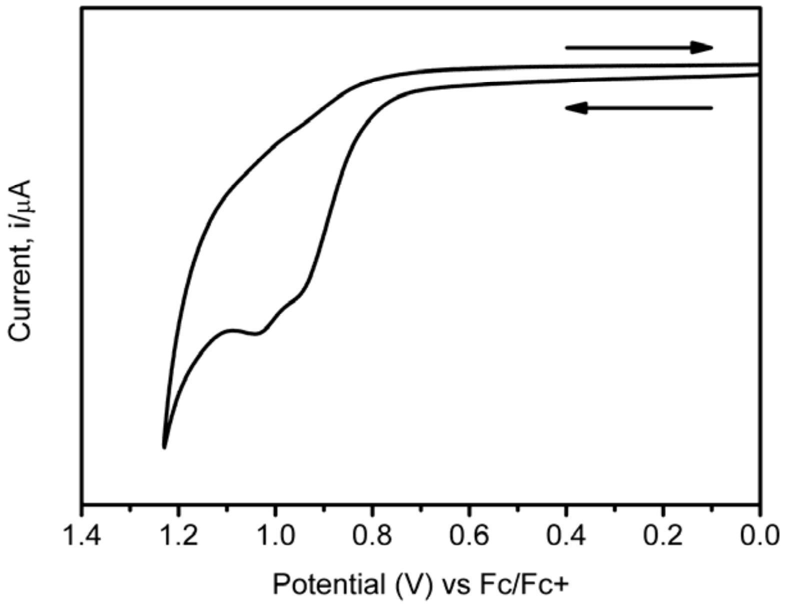

Supplementary Figure 16. Cyclic voltammagram of oxidative scan of **3Ph$_2$CzCzBN**.

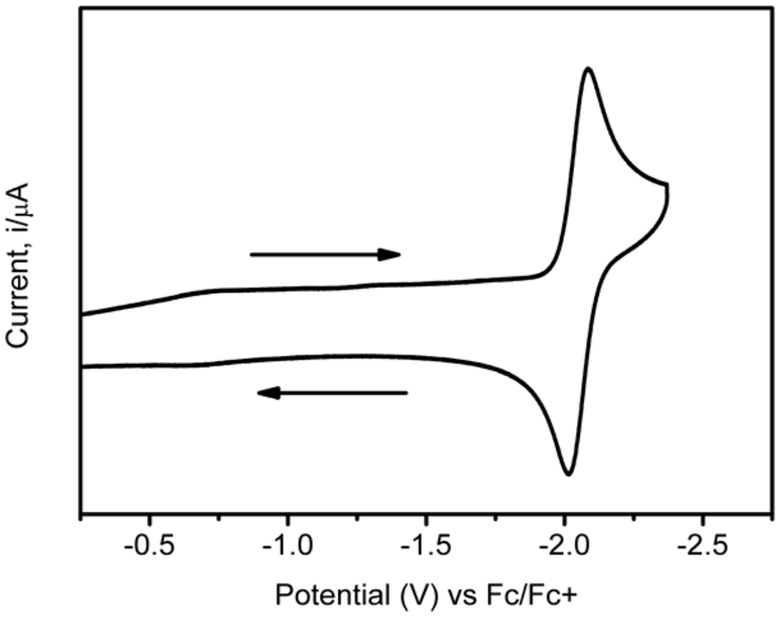

Supplementary Figure 17. Cyclic voltammagram of reductive scan of **3Ph$_2$CzCzBN**.

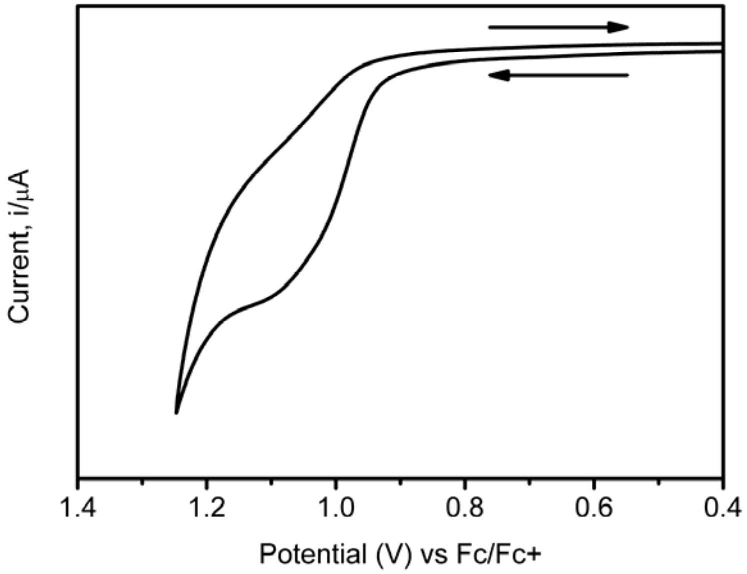

Supplementary Figure 18. Cyclic voltammagram of oxidative scan of **4CzBN**.

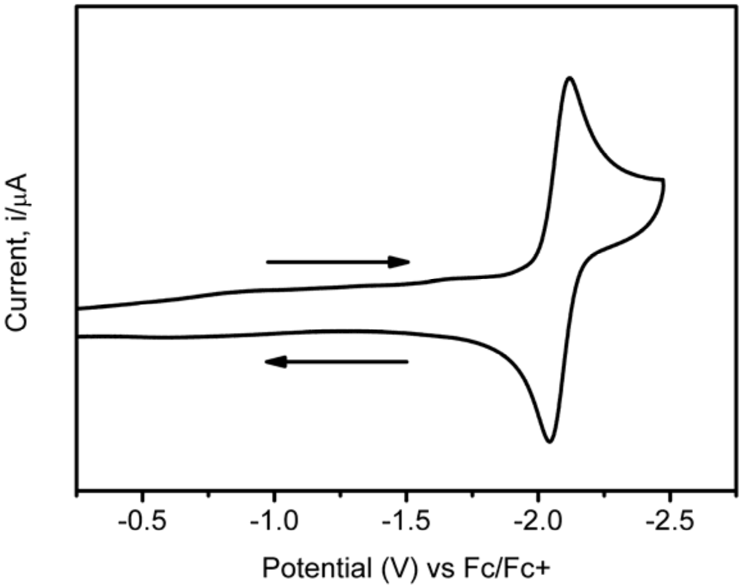

Supplementary Figure 19. Cyclic voltammagram of reductive scan of **4CzBN**.

Photoelectron spectroscopy

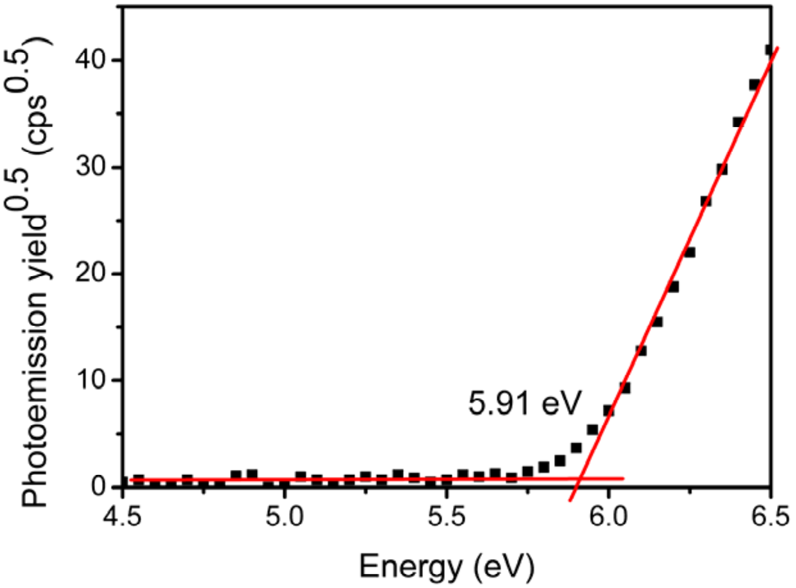

Supplementary Figure 20. Photoelectron spectrum of **3Ph$_2$CzCzBN** neat film by AC-3.

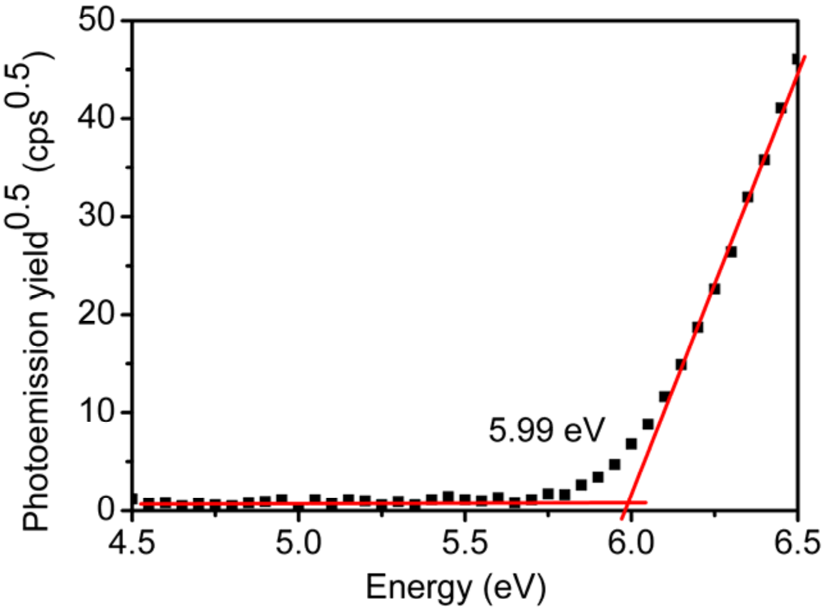

Supplementary Figure 21. Photoelectron spectrum of **4CzBN** neat film by AC-3.

Thermogravimetry - Differential thermal analysis (TG-DTA)

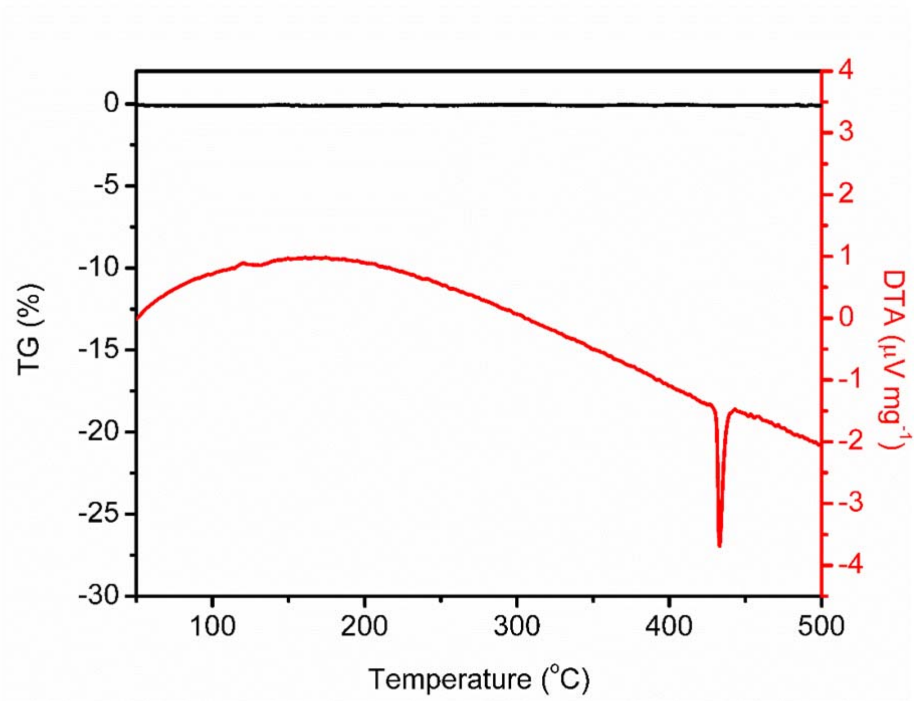

Supplementary Figure 22. TGA thermogram of **3Ph$_2$CzCzBN**.

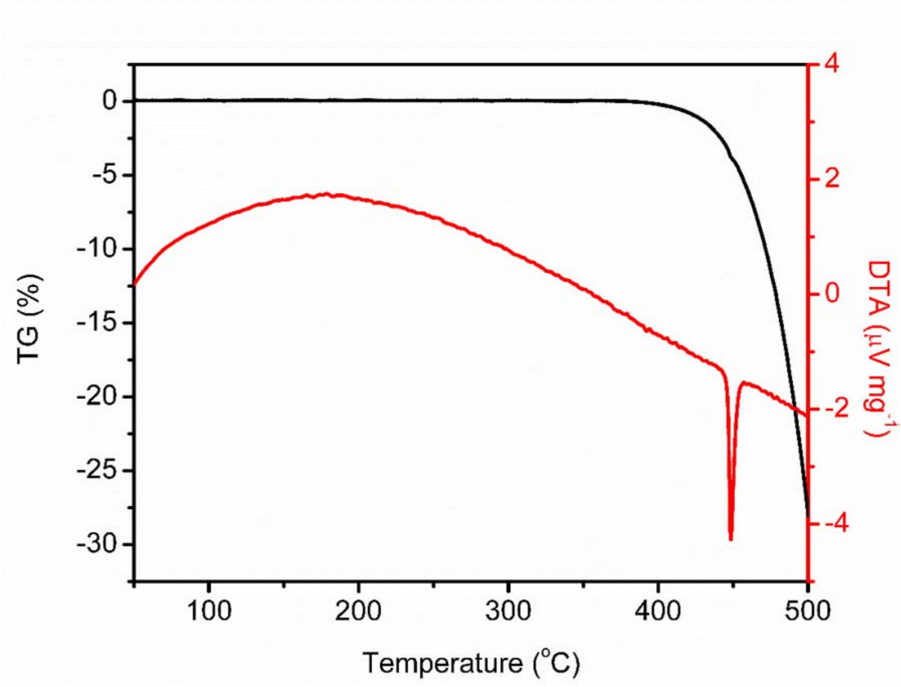

Supplementary Figure 23. TG-DTA analysis of **4CzBN**.

Differential scanning calorimetry (DSC)

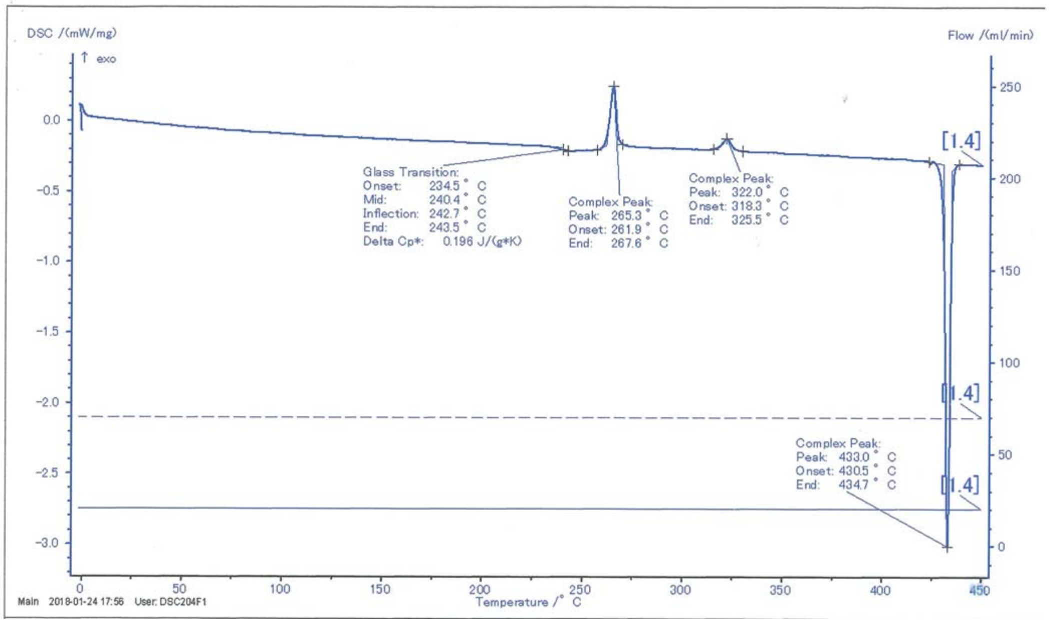

Supplementary Figure 24. DSC measurement of **3Ph$_2$CzCzBN**.

Device performance and stability of OLEDs

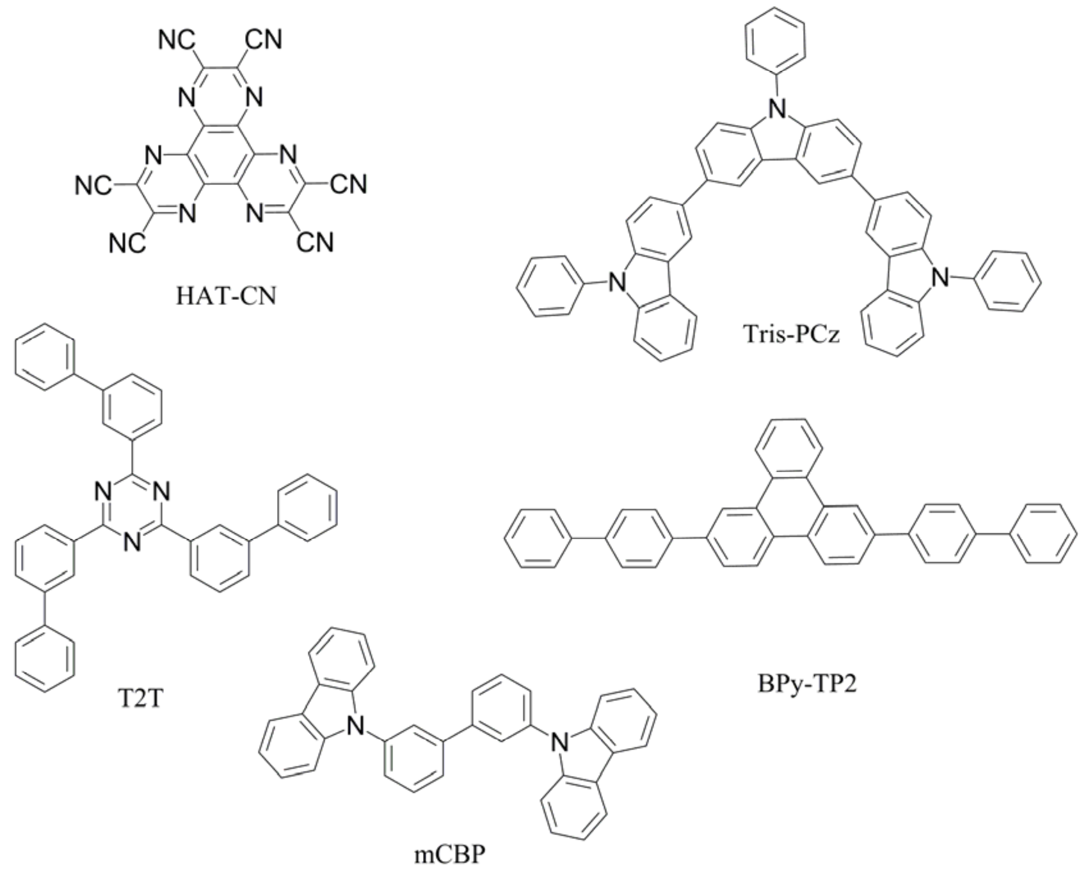

Supplementary Figure 25. The chemical structures of the materials used in Device A and B

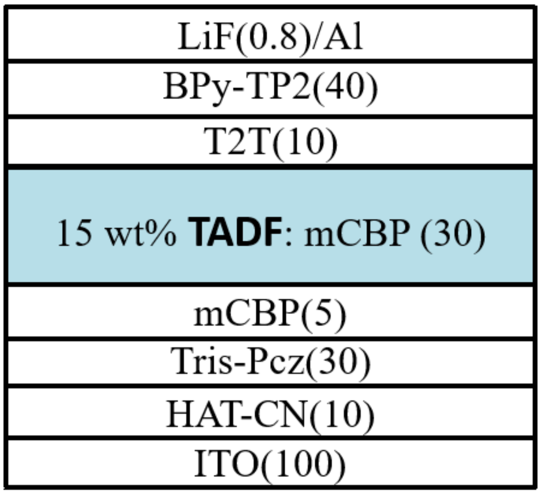

Supplementary Figure 26. Device structure for Device A and B.

Device A

ITO (100 nm)/ HAT-CN (10 nm)/ Tris-PCz (30 nm)/ mCBP (5 nm)/ 15 wt% of **3Ph2CzCzBN**: mCBP (30 nm)/ T2T (10 nm)/ BPy-TP2 (40 nm)/ LiF (0.8 nm)/ Al (100 nm)

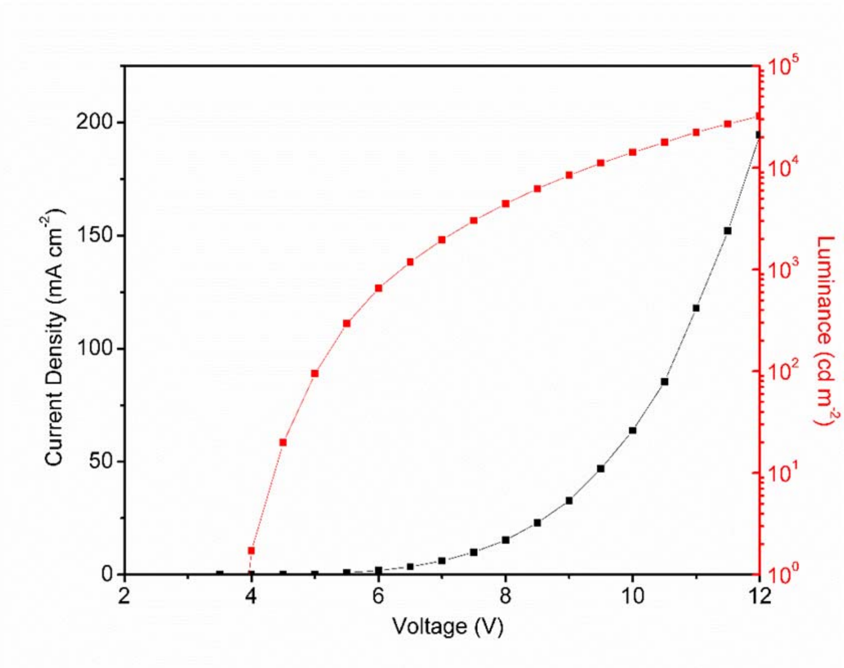

Supplementary Figure 27. Current density and luminance versus voltage.

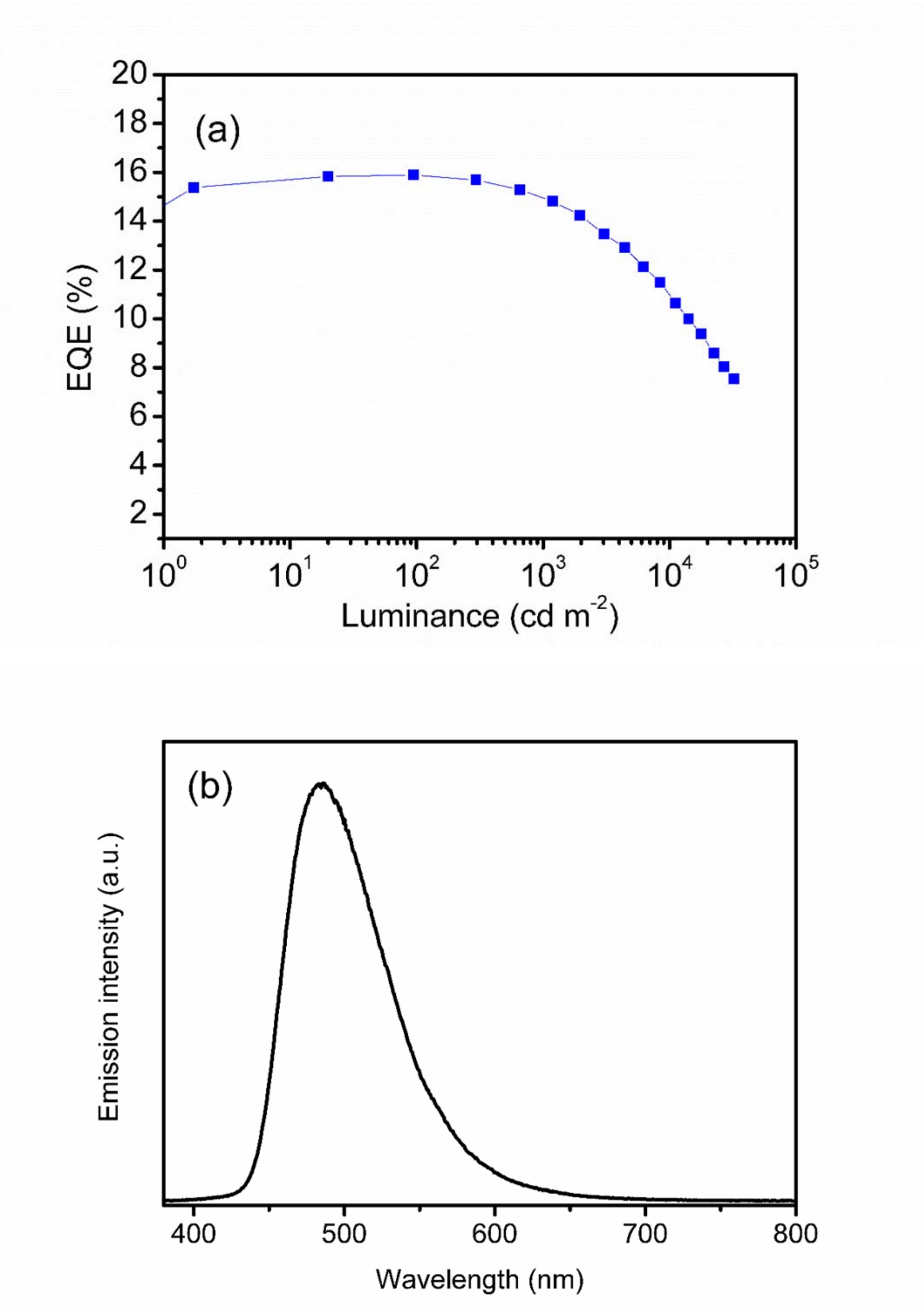

Supplementary Figure 28. a) EQE versus luminance; b) EL spectrum at 1000 cd m^{-2}.

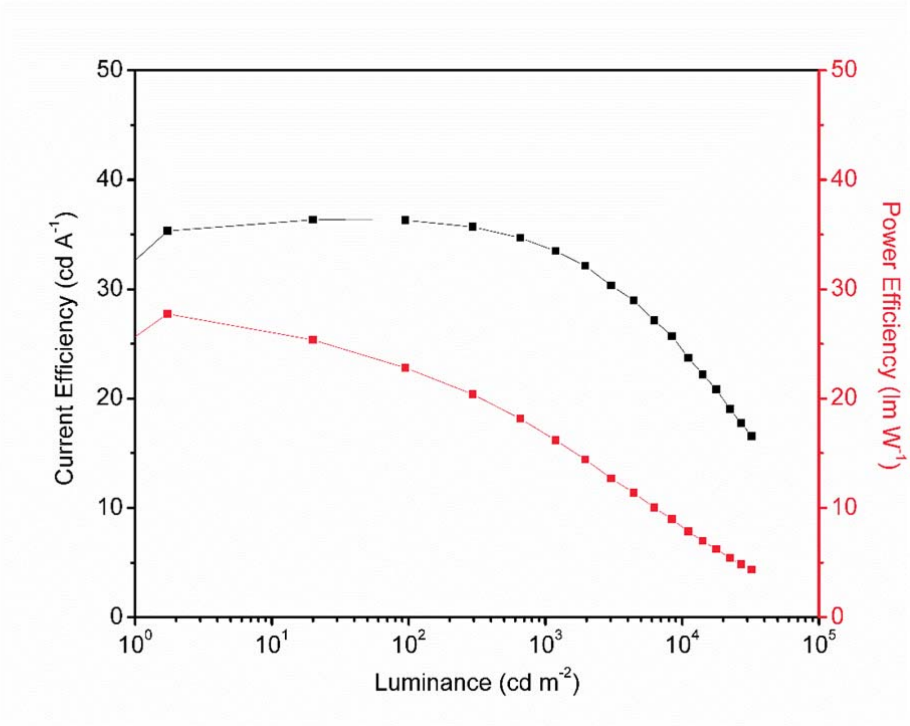

Supplementary Figure 29. Current efficiency and power efficiency versus luminance.

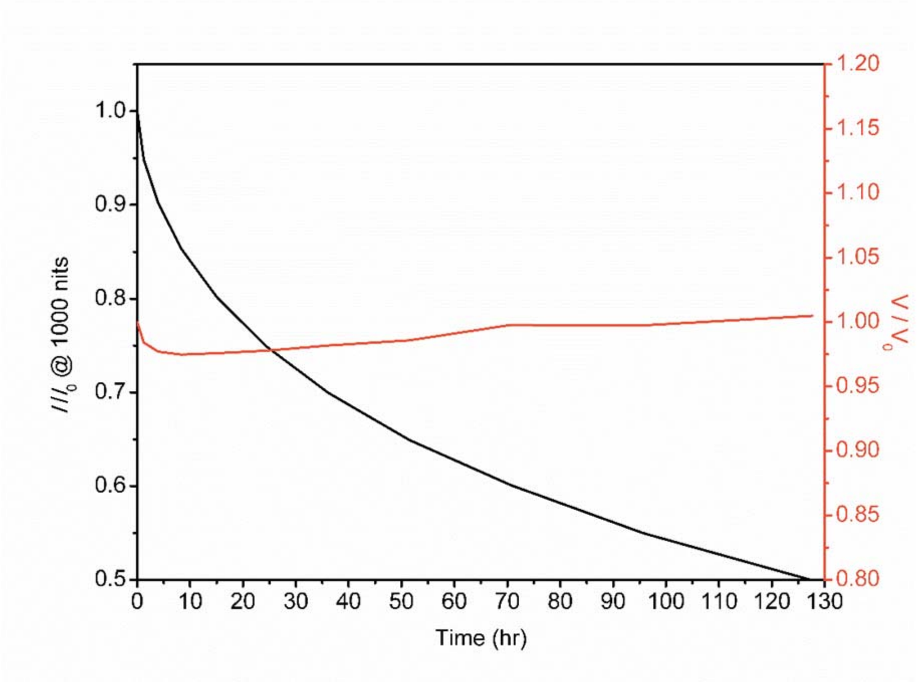

Supplementary Figure 30. Device lifetime at an initial luminance of 1000 cd m^{-2}.

Device B:

ITO (100 nm)/ HAT-CN (10 nm)/ Tris-PCz (30 nm)/ mCBP (5 nm)/ 15 wt% of **4CzBN**: mCBP (30 nm)/ T2T (10 nm)/ BPy-TP2 (40 nm)/ LiF (0.8 nm)/ Al (100 nm)

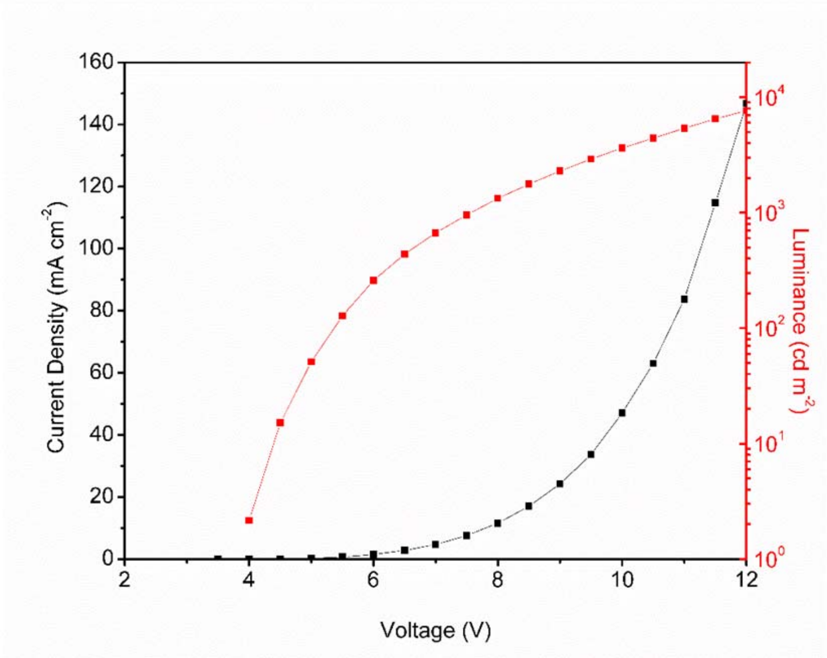

Supplementary Figure 31. Current density and luminance versus voltage.

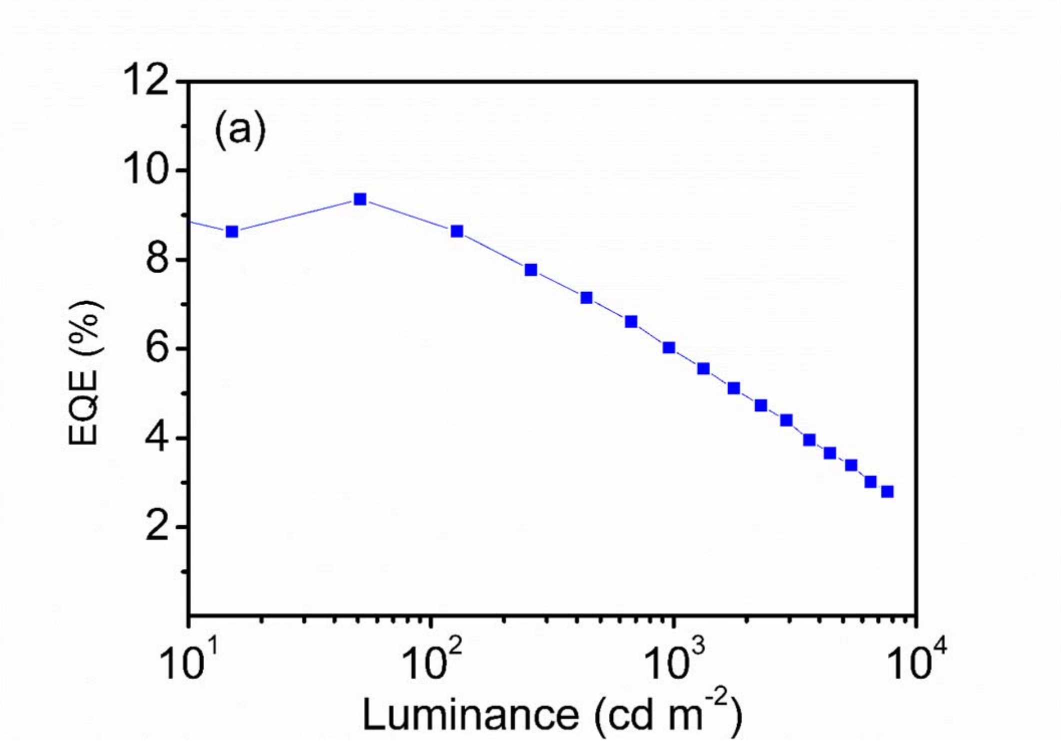

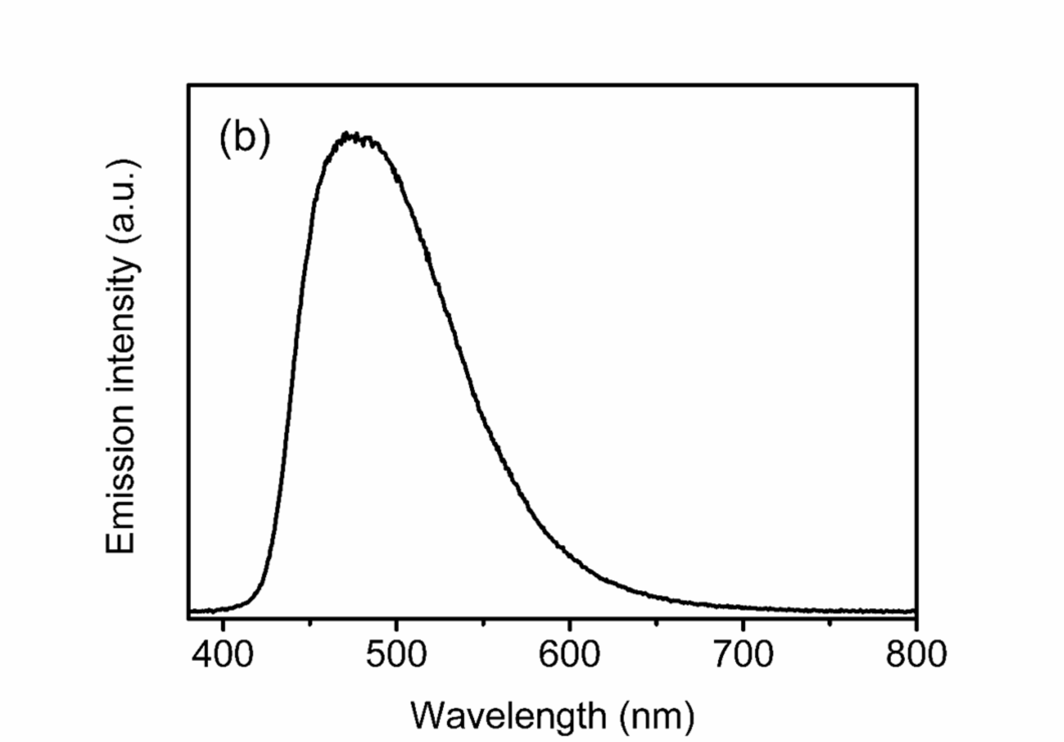

Supplementary Figure 32. a) EQE versus luminance; b) EL spectrum at 1000 cd m^{-2}.

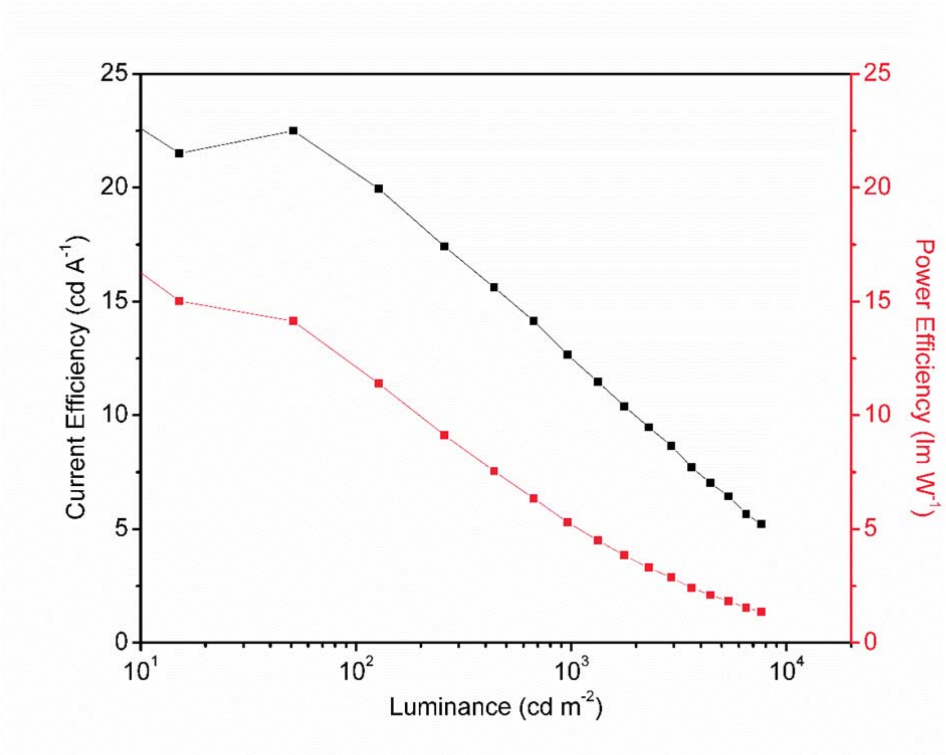

Supplementary Figure 33. Current efficiency and power efficiency versus luminance.

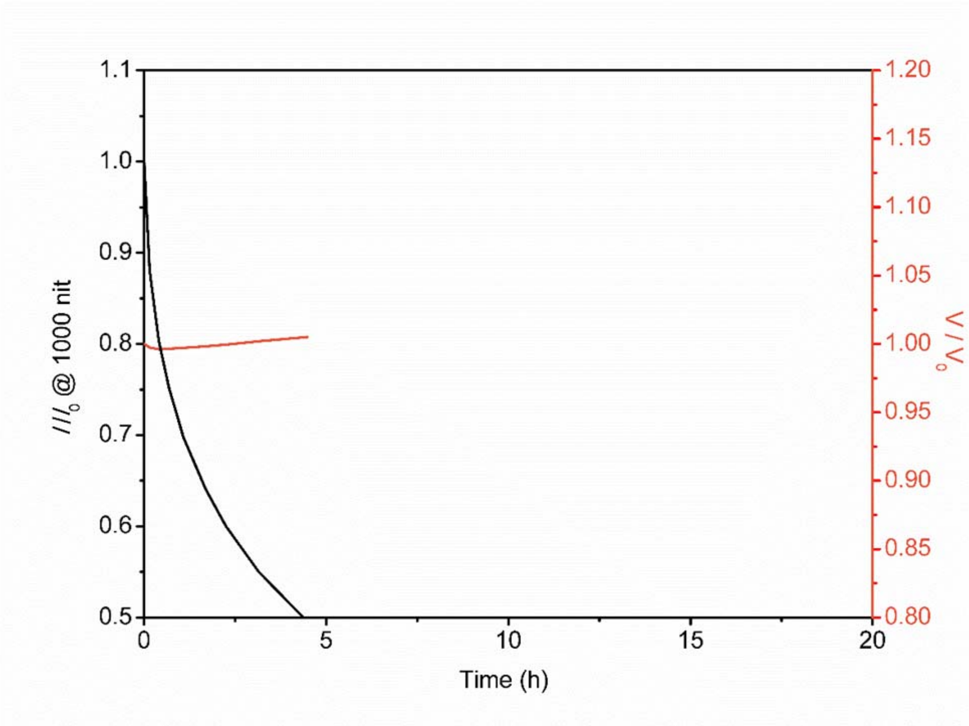

Supplementary Figure 34. Device lifetime at an initial luminance of 1000 cd m^{-2}.

Hole- and electron-only device

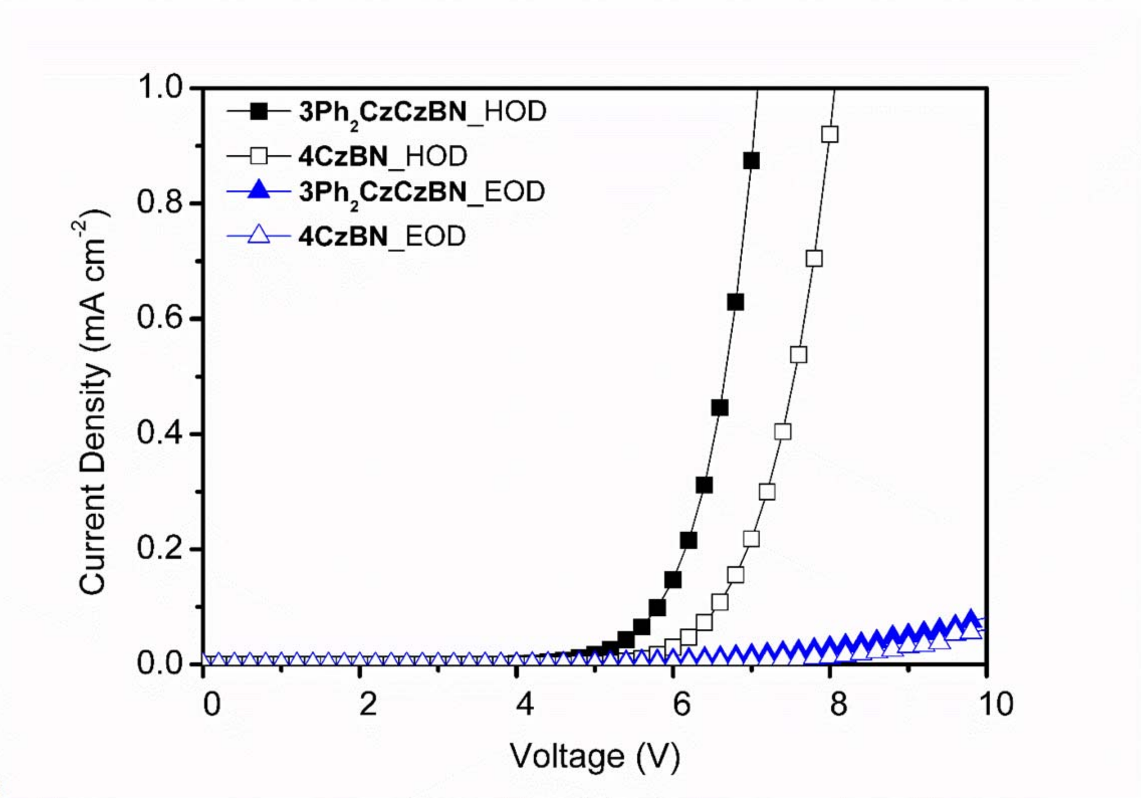

Supplementary Figure 35. Hole-only devices with the following configuration: ITO (100 nm)/HAT-CN(10 nm)/Tris-PCz(30 nm)/mCBP(5 nm)/15wt% TADF:mCBP(30 nm)/Tris-PCz(50 nm)/Al (100 nm)

Electron-only devices with the following configuration: ITO (100 nm)/T2T(30 nm)/ 15wt% TADF:mCBP(30 nm)/T2T (10 nm)/BPy-TP2(80 nm)/Al (100 nm)

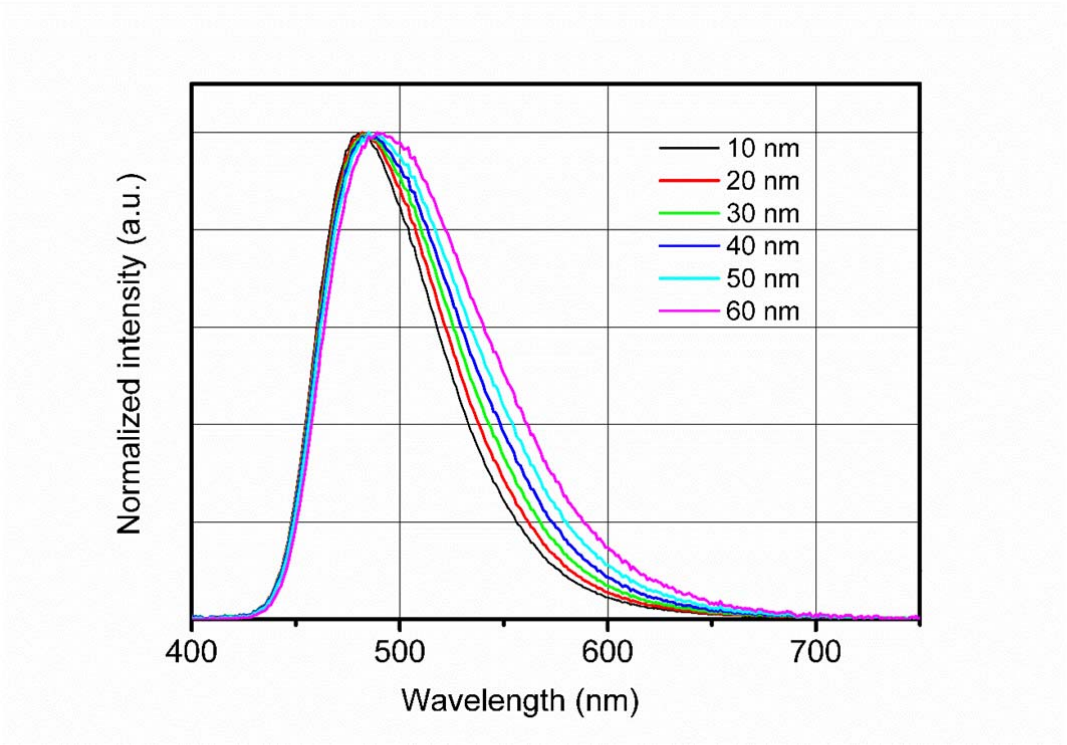

Supplementary Figure 36. Optical simulation of the EL spectra of the OLEDs based on device **D** with different thicknesses of the ETL. Device structure: ITO (100nm)/ HATCN (10 nm)/ Tris-PCz (30 nm)/ mCBP (5 nm)/ 20 wt% of 3Ph2CzCzBN : mCBP (30 nm)/ SF3-TRZ (10 nm)/ 30 wt % of Liq:SF3-TRZ **(10 to 60 nm)**/ Liq (2 nm)/ Al (100 nm).

Device **E**: ITO (100nm)/ HATCN (10 nm)/ Tris-PCz (30 nm)/ mCBP (5 nm)/ 20 wt% of **3Ph2CzCzBN** (device **D**): mCBP (30 nm)/ SF3-TRZ (10 nm)/ 30 wt % of Liq:SF3-TRZ (20 nm)/ Liq (2 nm)/ Al (100 nm).

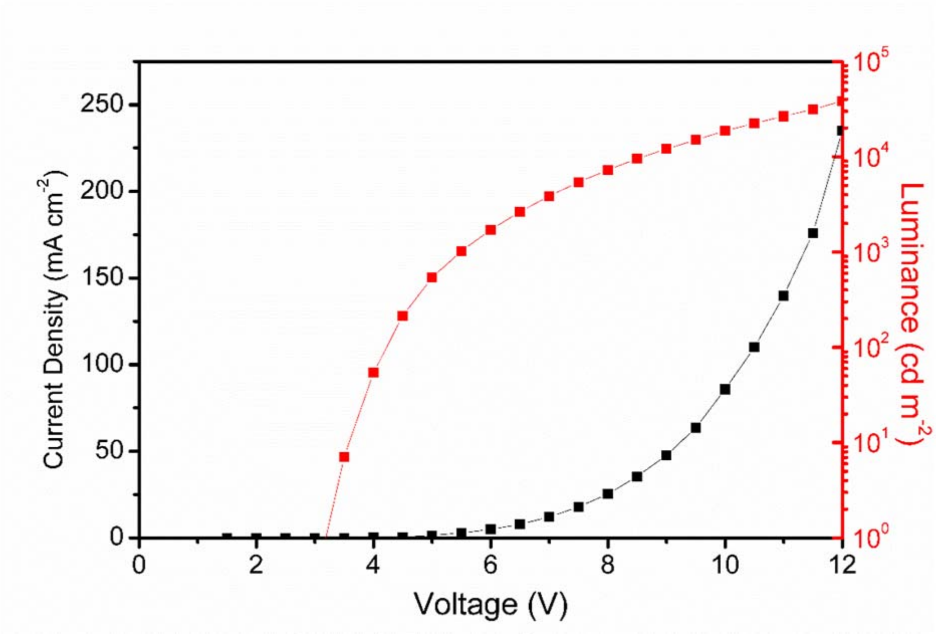

Supplementary Figure 37. Current density and luminance versus voltage.

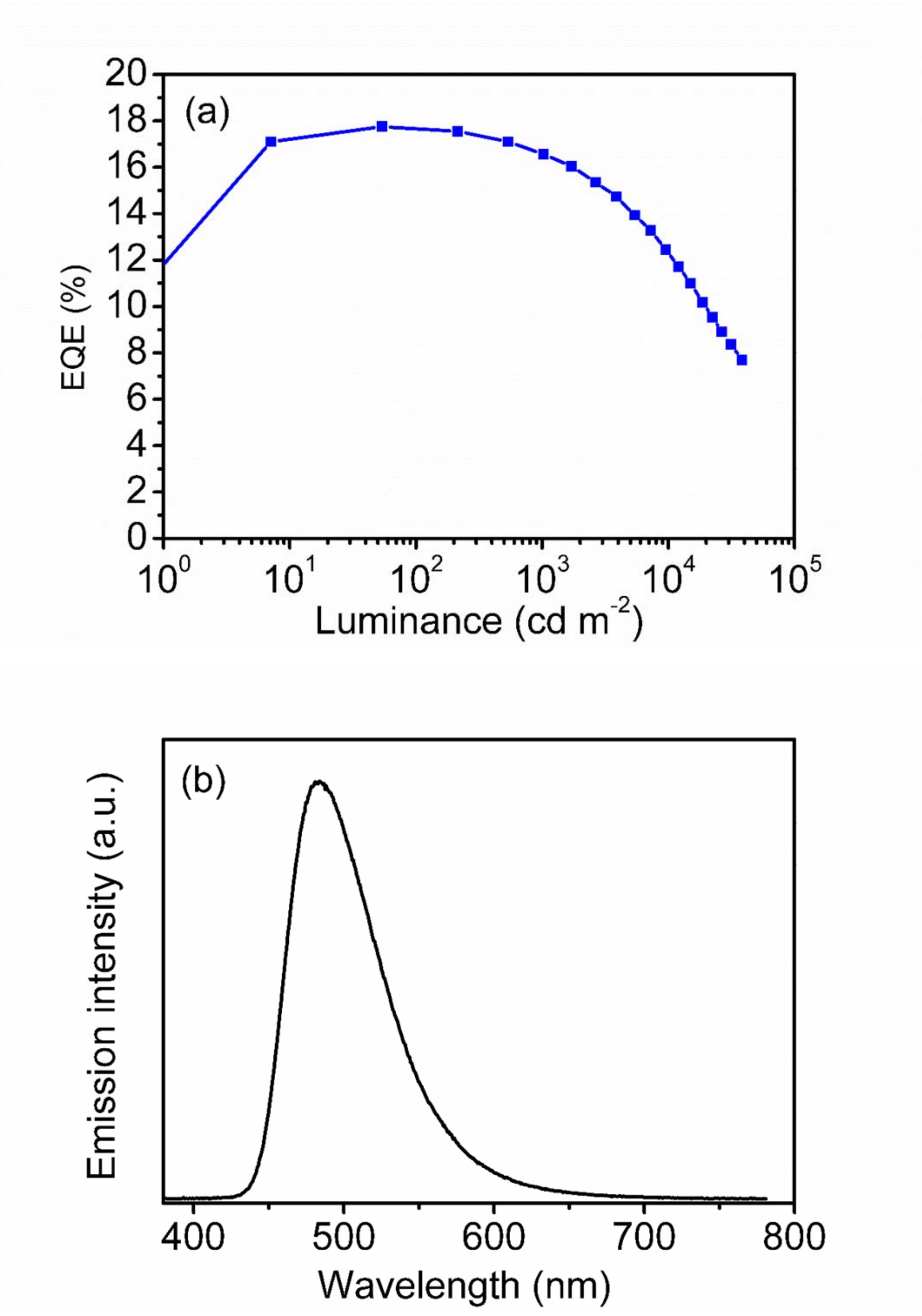

Supplementary Figure 38. a) EQE versus luminance; b) EL spectrum at 1000 cd m^{-2}.

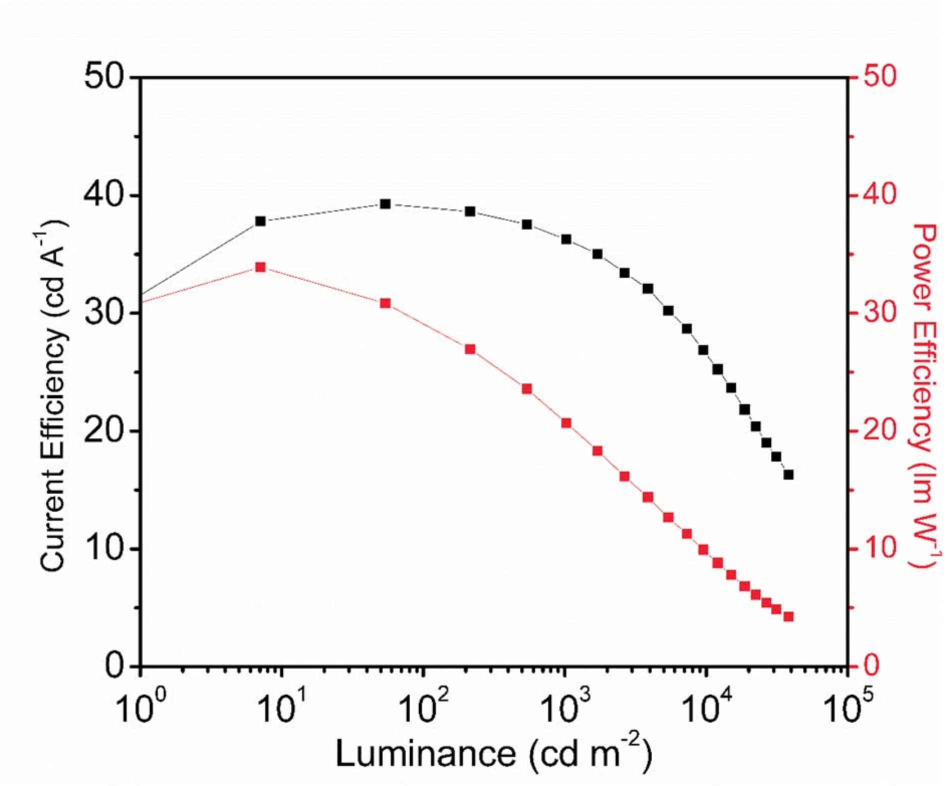

Supplementary Figure 39. Current efficiency and power efficiency versus luminance.

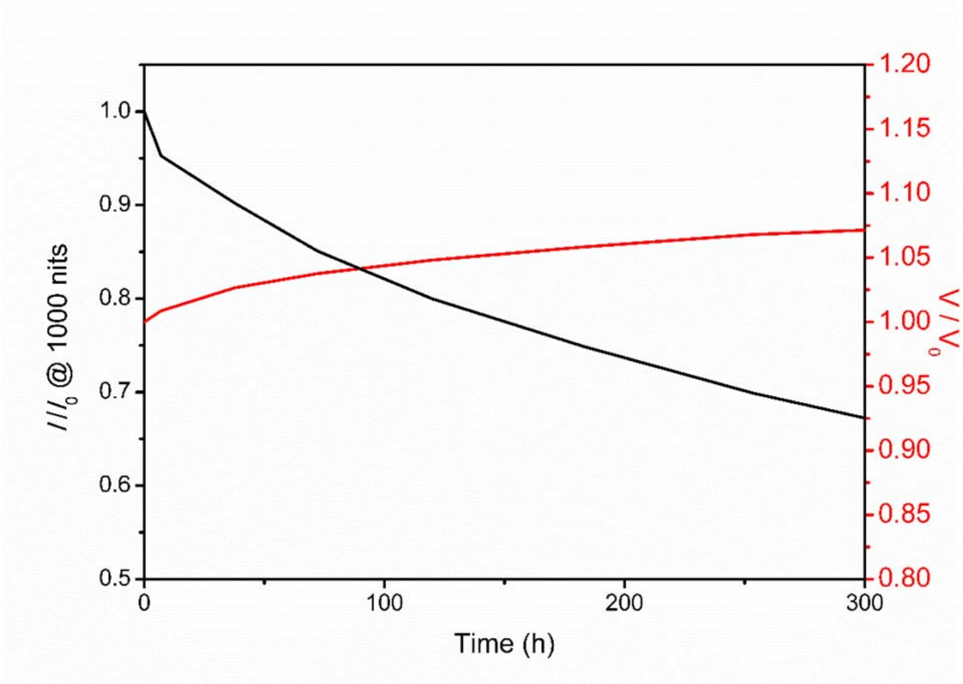

Supplementary Figure 40. Device lifetime at an initial luminance of 1000 cd m^{-2}.

Supplementary Table 1. Detailed photophysical properties and rate constants of 15

Compound	Film PLQY (%)	ϕ_p / ϕ_d (%)	Film τ_p (ns)	Film τ_d (μs)	$k_{r,S}/k_{nr,S}$ (10^8s)	k_{ISC} (10^8s)	k_{RISC} (10^5s)
**3Ph$_2$CzCzBN**	91	37/54	5.5	5.2	0.67/0.07	1.13	4.44
**4CzBN**	51	14/37	3.3	13.7	0.44/0.42	2.59	2.15

wt% doped film at 300K.

Supplementary Table 2. Detailed photophysical properties and rate constants of 15 wt % **3Ph$_2$CzCzBN** doped film from 200 to 300 K.

Temp (K)	ϕ_p (%)	ϕ_d(%)	k_p (10^8 s^{-1})	k_d (10^5 s^{-1})	k_{RISC} (10^5s)
300	37.1	53.9	1.81	1.93	4.44
290	31.1	46.3	1.81	1.74	3.75
280	30.7	46.8	1.79	1.61	3.53
270	29.6	46.6	1.79	1.50	3.36
260	28.9	46.4	1.79	1.38	3.11
250	28.4	46.0	1.75	1.28	2.90
240	27.6	45.0	1.75	1.19	2.68
230	28.2	45.4	1.72	1.10	2.45
220	27.8	43.9	1.73	1.01	2.21
210	27.9	42.3	1.72	0.95	2.00
200	26.6	39.3	1.64	0.92	1.85

Supplementary Table 3. Detailed photophysical properties and rate constants of 15 wt % **4CzBN** doped film from 200 to 300 K.

Temp (K)	ϕ_p (%)	ϕ_d(%)	k_p (10^8 s^{-1})	k_d (10^5 s^{-1})	k_{RISC} (10^5s)
300	14.4	36.6	3.03	0.73	2.15
290	14.0	35.8	2.81	0.61	1.82
280	14.0	34.3	2.83	0.54	1.53
270	13.9	33.2	2.57	0.47	1.31
260	13.9	30.0	2.65	0.42	1.05
250	13.9	27.2	2.74	0.39	0.88
240	14.3	24.8	2.63	0.36	0.74
230	14.1	21.7	2.81	0.33	0.60
220	14.1	19.0	2.93	0.33	0.51
210	14.1	15.5	2.85	0.31	0.40
200	14.4	13.4	2.67	0.27	0.30

Supplementary Table 4. Summary of some best reports on stable blue or sky-blue OLEDs with CIEy < 0.4.

Type[a]	Ref.	Emitter	EQE_{1000} (%)[b]	$Lum_{Initial}$ (cd m^{-2})[c]	V_{1000} (V)[d]	$EQE_{Initial}$ (%)[e]	LT_{90} (h)[f]	$CIE_{x,y}$
Phos	1	$Ir(dmp)_3$	8.0	1000	6.6	8.0	27	(0.15,0.28)
Phos	2	$Ir(dmp)_3$ (graded)	9.5	1000	7.7	9.5	~40	(0.16,0.33)
Phos	1	$Ir(dmp)_3$ (managed)	9.6	1000	9.0	9.6	141	(0.16,0.30)
Phos	3	$[Ir(mpmi)_2(pypz)]$	~9.0	950	-	~9.0	<1	(0.14, 0.27)
Phos	4	MS2	23.5	327	~6.8	~24	<1	(0.16,0.38)
Phos	5	FIrpic	≤5.1	400	–	≤5.1	<2	(0.15, 0.36)
Phos	5	$Ir(dfppy)_2$(pic-OH)	≤5.1	400	–	≤6.1	<2	(0.15, 0.35)
TADF	6	4CzBN	~4.0	500	~6.0	5.4	6	(0.17,0.20)
TADF	6	4TCzBN	~5.0	500	~6.0	6.5	6	(0.16,0.22)
TADF	7	BCz-TRZ	7.0	1000	5.8	7.0	~20	(0.20,0.36)
TADF	8	DDCzTrz	~2.0	500	8.0	~8.0	20	(0.16,0.24)
TADF	This work	**3Ph2CzCzBN (device C)**	14.3	1000	6.4	14.3	16	(0.18,0.37)
TADF	This work	**3Ph2CzCzBN (device D)**	17.2	1000	5.8	17.2	32	(0.18,0.39)
TADF	This work	**3Ph2CzCzBN (device E)**	16.6	1000	5.5	16.6	38	(0.17,0.36)

[a]Phos = Phosphorescence; TADF = Thermally activated delayed fluorescence. [b]EQE at 1000 cd m^{-2}. [c]Initial luminance for testing device stability. [d]Driving voltage at 1000 cd m^{-2}. [e]EQE at the initial luminance for testing device stability. [f]90 % of the initial luminance.

Supplementary Methods

Synthesis

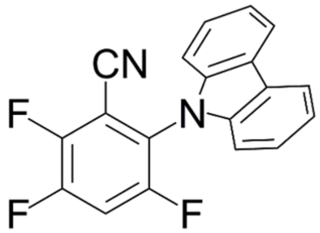

2-(9H-Carbazol-9-yl)-3,5,6-trifluorobenzonitrile

Under nitrogen atmosphere, 9*H*-carbazole (167 mg, 1 mmol) was dissolved in dry *N*,*N*-dimethylformamide (30 mL) in a two-neck round-bottom flask equipped with a condenser. The reaction mixture was cooled to 0 °C, then NaH (40 mg, 1 mmol) was added. The reaction mixture was slowly warmed to room temperature and stirred for half an hour. After that, tetrafluorobenzonitrile (175 mg, 1 mmol) was added and the reaction was stirred at room temperature for 16 hours. The reaction was quenched with water and the precipitate was filtered off. The crude product was purified by column chromatography. Yield: 193 mg (60%). ^{1}H NMR (500 MHz, $CDCl_3$, 298 K, relative to Me_4Si): δ = 8.16 (d, 2H, 7.5 Hz), 7.53-7.58 (m, 1H), 7.48 (t, 2H, 7.0 Hz), 7.38 (t, 2H, 7.5 Hz), 7.11 (d, 2H, 8.0 Hz); ^{13}C NMR (126 MHz, $CDCl_3$): δ = 155.8, 155.7, 153.8, 153.7, 150.9, 150.8, 150.7, 150.1, 150.0, 149.9, 148.8, 148.7, 148.6, 148.0, 147.9, 147.8, 140.0, 126.6, 124.4, 124.3, 124.3, 124.2, 124.2, 121.6, 120.8, 112.2, 112.0, 111.9, 111.8, 109.4, 109.3, 109.2,109.2, 105.5, 105.4; ^{19}F NMR (471 MHz, $CDCl_3$): δ = -114.32 (m, 1H), -128.05 (m, 1H), -130.54 (m, 1H); MS (APCI) calcd. for $C_{19}H_9F_3N_2$: *m/z* = 322.08; found: 322.17 $[M]^+$.

Supplementary Notes

Rate Constants

The k_{RISC} is calculated according to the reported method.[9,10] By assuming $k_{\mathrm{nr,S}} << k_{\mathrm{r,S}}$, k_{ISC} and $k_{\mathrm{p}} >> k_{\mathrm{d}}$, the corresponding rate constants can be calculated with the following equations:

$$k_{r,S} = \phi_p k_p + \phi_d k_d \approx \phi_p k_p$$

$$k_{ISC} \approx k_p(1 - \phi_p)$$

$$k_{nr,S} = k_{r,S}\frac{(1-\phi)}{\phi}$$

$$k_{RISC} \approx \frac{k_p k_d \phi_d}{k_{ISC}\phi_p}$$

Device A and B

Indium–tin oxide (ITO)-coated glass (100 nm)/ HAT-CN (10 nm)/ Tris-PCz (30 nm)/ mCBP (5 nm)/ 15 wt% of **3Ph2CzCzBN** (device **A**) or 15 wt% of **4CzBN** (device **B**): mCBP (30 nm)/ T2T (10 nm)/ BPy-TP2 (40 nm)/ LiF (0.8 nm)/ Al (100 nm), iin which ITO is the anode, 1,4,5,8,9,11-hexaazatriphenylene-hexacarbonitrile (HATCN) is the hole-injection layer, 9,9',9''-triphenyl-9H,9'H,9''H-2,3':6',2''-tercarbazole (Tris-PCz) is the hole-transporting layer, 3,3′-di(9H-carbazol-9-yl)-1,1′-biphenyl (mCBP) was the exciton-blocking layer, 15 wt% of **3Ph2CzCzBN** (device **A**) or 15 wt% of **4CzBN** (device **B**) doped in mCBP was the emitting layer (EML), 2,4,6-tris(biphenyl-3-yl)-1,3,5-triazine (T2T) was the exciton-blocking layer, 2,7-di(2,2'-bipyridin-5-yl)triphenylene (BPy-TP2) was the electron-transporting layer and LiF and Al were the electron injection layer and cathode, respectively.

Supplementary References

1.

2.

3. Lee, J. et al. Hot excited state management for long-lived blue phosphorescent organic light-emitting diodes. *Nat. Commun.* **8**, 15566 (2017).
4. Zhang, Y., Lee, J. & Forrest, S. Tenfold increase in the lifetime of blue phosphorescent organic light-emitting diodes. *Nat. Commun.* **5**, 5008 (2014).
5. Hsieh, C.-H. et al. Design and synthesis of iridium bis(carbine) complexes for efficient blue electrophosphorescence. *Chem. Eur. J.* **17**, 9180-9187 (2011).
6. Sarma, M. et al. Anomalously long-lasting blue PhOLED featuring phenyl-pyrimidine cyclometalated iridium emitter. *Chem* **3**, 461-476 (2017).
7. Yi, S. et al. Stable blue phosphorescence iridium(III) cyclometalated complexes prompted by intramolecular hydrogen bond in ancillary ligand. *Inorg. Chem*. **55**. 3324-3331 (2016).
8. Zhang, D., Cai, M., Zhang, Y., Zhang, D. & Duan, L. Sterically shielded blue thermally activated delayed fluorescence emitters with improved efficiency and stability. *Mater. Hori*. **3**, 145-151 (2016).
9. Cui, L.-S. et al. Long-lived efficient delayed fluorescence organic light-emitting diodes using n-type hosts. *Nat. Commun.* **8**, 2250 (2017).
10. Kim, M., Jeon, S. K., Hwang, S.-H. & Lee, J. Y. Stable blue thermally activated delayed fluorescent organic light-emitting diodes with three times longer lifetime than phosphorescent organic light-emitting diodes. *Adv. Mater.* **27**, 2515-2520 (2015).

11. Goushi, K., Yoshida, K., Sato, K. & Adachi, C. Organic light emitting-diodes employing efficient reverse intersystem crossing for triplet-singlet state conversion. *Nat. Photon*. **6**, 253-258 (2012).

12. Pan, K.-C. et al. Efficient and tunable thermally activated delayed fluorescence emitters having orientation - adjustable CN - substituted pyridine and pyrimidine acceptor units. *Adv. Funct. Mater.* **26**, 7560-7571 (2017).
